# Societal impact for patients with psoriasis: A nationwide Swedish register study

**DOI:** 10.1016/j.jdin.2021.02.003

**Published:** 2021-03-30

**Authors:** Henrike Häbel, Björn Wettermark, David Hägg, Reginald Villacorta, E.Christina M. Wennerström, Marie Linder

**Affiliations:** a Unit of Biostatistics, Institute of Environmental Medicine, Karolinska Institutet, Stockholm, Sweden; b Department of Pharmacy, Disciplinary Domain of Medicine and Pharmacy, Uppsala University, Uppsala, Sweden; c Centre for Pharmacoepidemiology, Department of Medicine, Karolinska Institutet, Stockholm, Sweden; d Janssen Pharmaceuticals, Brunnswick, New Jersey; e Janssen-Cilag, Solna, Sweden; f Department of Epidemiology Research, Statens Serum Institut, Copenhagen, Denmark

**Keywords:** disease burden, drug utilization, psoriasis, societal impact, ICD, International Classification of Diseases, NPR, National Patient Register, PDR, Swedish Prescribed Drug Register, SEK, Swedish crowns, TPR, Total Population Register

## Abstract

**Background:**

Psoriasis is an immune-mediated chronic inflammatory disease having a significant negative health impact. Psoriasis has societal impact; loss of productivity has been estimated at approximately 10% and it may influence the patient's financial status. Relationships between quality of life, disease severity, and cost of care need exploration. Understanding the disease burden is important for health policy and research allocation. Few studies address the research gaps in socioeconomics, comorbidity, and medication use.

**Objective:**

Observing differences in education, income, employment status, marital status, health care consumption, and drug utilization between patients with psoriasis and matched controls.

**Methods:**

Cohort study following socioeconomics and health care consumption for all psoriasis patients from the Swedish patient register. All individuals with a first diagnosis of psoriasis in outpatient or inpatient care from 2002 to 2013 were followed until death, emigration, or end of the study.

**Results:**

Overall, 109,803 patients were included (mean age 51.2 years, 53% women) and matched with 1.08 million controls. The levels of education and income were similar, but the proportion employed was significantly lower for patients with psoriasis. There was a tendency for fewer patients with psoriasis to be married.

**Limitations:**

Generalizability, lack of primary care diagnoses, and lack of early treatments (available from 2005).

**Conclusion:**

Understanding of the socioeconomic impact of psoriasis is extended by showing reductions in employment.

**Capsule Summary**

• Psoriasis has a negative health impact and increases community resource use and health care consumption. Our study suggests lower societal productivity by reductions in employment, despite similar incomes to controls.
• There is need for long-term monitoring of patients with psoriasis shown by continued increased health care utilization as compared with controls.

## Introduction

Psoriasis is a common immune-mediated chronic inflammatory disease with worldwide impact on men and women of all ages, affecting 2%-4% of the Nordic population,^1^^,^^2^ corresponding to 200,000-400,000 individuals in Sweden. Psoriasis has a negative health impact and increases community resource use and health care consumption. Studies have shown societal impact; global loss of productivity among psoriasis patients was estimated to approximately 10%.^3^ Other studies have reported that patients with psoriasis lose 2.3 working days per month^4^ or 3 days per year in sick leave.^3^ In a review of studies published between January 2001 and May 2013, the economic burden of psoriasis was reported in 35 studies (11 countries).^5^ The most recent studies reported an annual cost per patient of €11,928 in Sweden, €8,372 in Italy, and €2,866-€6,707 in Germany. Some costs were related to health care and others to sick leave and disability. Even though there are substantial costs associated with psoriasis, costs can be reduced with treatment.^6^

Though there are limited studies on patterns of health care consumption and sick leave, a Swedish study in patients with ankylosing spondylitis and psoriatic arthritis showed that sickness absences declined by 11.7 days for women and 7.6 days for men once psoriasis was diagnosed.^7^ Patients with psoriasis also have more comorbidities, such as diabetes and hypertension, impacting work ability and lifestyle.^8^^,^^9^ A focus group in patients with psoriasis revealed that the disease has implications on health care consumption as a consequence of its impact on mental health and well-being.^10^ Besides medical symptoms, psoriasis may influence a patient's income and finances.^8^^,^^9^

Psoriasis prevalence has been relatively stable since the mid-2000s,^11^ and studies have demonstrated that patients with severe disease have an increased mortality risk.^12^^,^^13^ Understanding the disease burden is important for health policy and research allocation to improve patient outcomes. A number of studies have investigated cause-specific mortality in severe psoriasis, suggesting an association with the increased risk of stroke, cardiovascular disease, and other comorbid conditions.^13^, ^14^, ^15^, ^16^, ^17^ In a previous Swedish study, patients with severe psoriasis were reported to have higher overall mortality and higher cardiovascular disease risk, which is a strong determinant of excess mortality.^18^ Few studies addressed the research gaps socioeconomics, comorbidities, and medication use over time and to what extent there are disparities related to the degree of disease control.^19^

The objectives of our study were to estimate and compare differences in education, income, employment, and marital status between patients with psoriasis and controls. Further, our study described and compared health care and drug utilization.

## Materials and methods

### Study design

**Fig 1:**
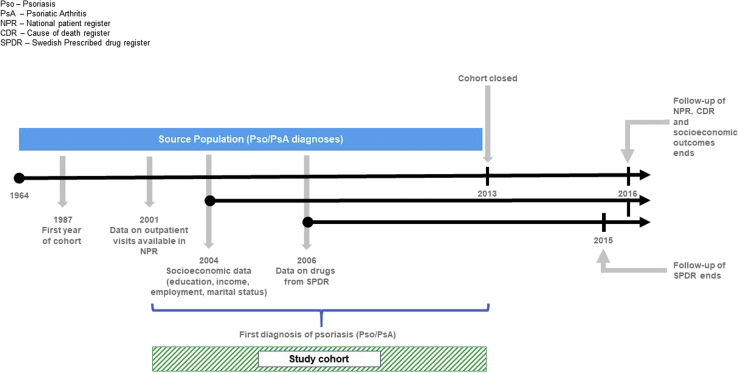
Psoriasis study design (adapted from Schneeweiss S, Rassen JA, Brown JS, et al. Graphical depiction of longitudinal study designs in health care databases. *Ann Intern Med*. 2019;170(6):398-406. https://doi.org/10.7326/M18-3079).

This longitudinal, matched cohort study included all adult patients (>17 years) with a diagnosis of psoriasis who were identified through national Swedish health registers between 1987 and 2013, refers to the date of diagnosis. The study design is illustrated in Fig 1 and described following.

### Setting

The study was conducted in Sweden, a country of 10 million inhabitants with a comprehensive health care system that aims to ensure everyone's equal access to health care.^20^ Swedish health care is decentralized to 21 regions and regulated by the Health and Medical Service Act. The high degree of self-governance following budget devolution, political majorities, and sociodemographic demands and structures have resulted in regional differences in quality of care, but efforts were taken to strengthen joint work.^21^ The National Board of Health and Welfare have issued guidelines for psoriasis,^22^ aimed at reducing regional differences, that contain recommendations around living habits, complicity, investigation, follow-up, and treatments.

### Data sources

Data from Swedish national registers were linked through the unique personal identity number.^23^ Information on diagnoses, procedures, hospitalizations, and outpatient consultations in specialist care were collected from the National Patient Register (NPR) between 1987 and 2016 (Fig 1).^24^ Inpatient data were available from 1987 and outpatient data from 2001. Diagnoses between 1987 and 1997 were recorded using International Classification of Diseases (ICD), 9th revision; diagnoses between 1997 and the current time were recorded using ICD, 10th revision (Table I). Information on drugs was collected from the Swedish Prescribed Drug Register (PDR) starting in July 2005.^25^ The Swedish PDR contains dispensed items, amount, and date of all filled prescriptions. Drugs were recorded using the Anatomical Therapeutic Chemical classification system.^26^ Information on deaths were collected from the Cause of Death Register.^27^ Migrations and county of residence were retrieved between 2005 and 2016 from the Total Population Register (TPR). Education, income, employment status, and marital status were obtained from the longitudinal integration database for health insurance and labor market studies,^28^ between 2004 and 2016. These socioeconomic variables are available only on a yearly basis.

**Table I:** International Classification of Diseases, 10^th^ revision (ICD10) codes for identification of comorbidities

Comorbidity	Text	ICD10
Myocardial infarction incl. congestive heart failure	Acute myocardial infarction	I21
	Subsequent myocardial infarction	I22
	Old myocardial infarction	I252
	Cardiomyopathy in diseases classified elsewhere	I43
	Heart failure	I50
	Rheumatic heart disease, unspecified	I099
	Hypertensive heart disease with (congestive) heart failure	I110
	Hypertensive heart and renal disease with (congestive) heart failure	I130
	Hypertensive heart and renal disease with both (congestive) heart failure and renal failure	I132
	Ischemic cardiomyopathy	I255
	Dilated cardiomyopathy	I420
	Other restrictive cardiomyopathy	I425
	Alcoholic cardiomyopathy	I426
	Cardiomyopathy due to drugs and other external agents	I427
	Other cardiomyopathies	I428
	Cardiomyopathy, unspecified	I429
Peripheral vascular disease	Atherosclerosis	I70
	Aortic aneurysm and dissection	I71
	Thromboangiitis obliterans [Buerger]	I731
	Other specified peripheral vascular diseases	I738
	Peripheral vascular disease, unspecified	I739
	Stricture of artery	I771
	Aneurysm of aorta in diseases classified elsewhere	I790
	Peripheral angiopathy in diseases classified elsewhere	I792
	Chronic vascular disorders of intestine	K551
	Other vascular disorders of intestine	K558
	Vascular disorder of intestine, unspecified	K559
	Presence of other cardiac and vascular implants and grafts	Z958
	Presence of cardiac and vascular implant and graft, unspecified	Z959
Cerebrovascular disease	Transient cerebral ischemic attacks and related syndromes	G45
	Vascular syndromes of brain in cerebrovascular diseases	G46
	Subarachnoid hemorrhage	I60
	Intracerebral hemorrhage	I61
	Other nontraumatic intracranial hemorrhage	I62
	Cerebral infarction	I63
	Stroke, not specified as hemorrhage or infarction	I64
	Occlusion and stenosis of precerebral arteries, not resulting in cerebral infarction	I65
	Occlusion and stenosis of cerebral arteries, not resulting in cerebral infarction	I66
	Other cerebrovascular diseases	I67
	Cerebrovascular disorders in diseases classified elsewhere	I68
	Sequelae of cerebrovascular disease	I69
	Transient retinal artery occlusion	H340
Chronic obstructive pulmonary disease	Bronchitis, not specified as acute or chronic	J40
	Simple and mucopurulent chronic bronchitis	J41
	Unspecified chronic bronchitis	J42
	Emphysema	J43
	Other chronic obstructive pulmonary disease	J44
	Asthma	J45
	Status asthmaticus	J46
	Bronchiectasis	J47
	Coalworker pneumoconiosis	J60
	Pneumoconiosis due to asbestos and other mineral fibres	J61
	Pneumoconiosis due to dust containing silica	J62
	Pneumoconiosis due to other inorganic dusts	J63
	Unspecified pneumoconiosis	J64
	Pneumoconiosis associated with tuberculosis	J65
	Airway disease due to specific organic dust	J66
	Hypersensitivity pneumonitis due to organic dust	J67
	Other specified pulmonary heart diseases	I278
	Pulmonary heart disease, unspecified	I279
	Chronic respiratory conditions due to chemicals, gases, fumes and vapors	J684
	Chronic and other pulmonary manifestations due to radiation	J701
	Chronic drug-induced interstitial lung disorders	J703
Dementia	Dementia in Alzheimer disease	F00
	Vascular dementia	F01
	Dementia in other diseases classified elsewhere	F02
	Unspecified dementia	F03
	Alzheimer disease	G30
	Senile degeneration of brain, not elsewhere classified	G311
	Delirium superimposed on dementia	F051
Diabetes (incl. complications)	Type 1 diabetes mellitus	E10
	Type 2 diabetes mellitus	E11
	Malnutrition-related diabetes mellitus	E12
	Other specified diabetes mellitus	E13
	Unspecified diabetes mellitus	E14
Peptic ulcers	Gastric ulcer	K25
	Duodenal ulcer	K26
	Peptic ulcer, site unspecified	K27
	Gastrojejunal ulcer	K28
Rheumatic disease	Seropositive rheumatoid arthritis	M05
	Other rheumatoid arthritis	M06
	Systemic lupus erythematosus	M32
	Dermatopolymyositis	M33
	Systemic sclerosis	M34
	Giant cell arteritis with polymyalgia rheumatica	M315
	Other overlap syndromes	M351
	Polymyalgia rheumatica	M353
	Dermato(poly)myositis in neoplastic disease	M360
Cancer and metastatic solid tumor, excluding non-melanoma skin cancer	Malignant neoplasms, stated or presumed to be primary, of specified sites, except of lymphoid, hematopoietic and related tissue	C00-C43
	Malignant neoplasms, stated or presumed to be primary, of specified sites, except of lymphoid, hematopoietic and related tissue	C45-C75
	Malignant neoplasms of ill-defined, secondary and unspecified sites	C76-C80
	Malignant neoplasms, stated or presumed to be primary, of lymphoid, hematopoietic and related tissue	C81-C96
	Malignant neoplasms of independent (primary) multiple sites	C97-C97

### Study population

**Fig 2:**
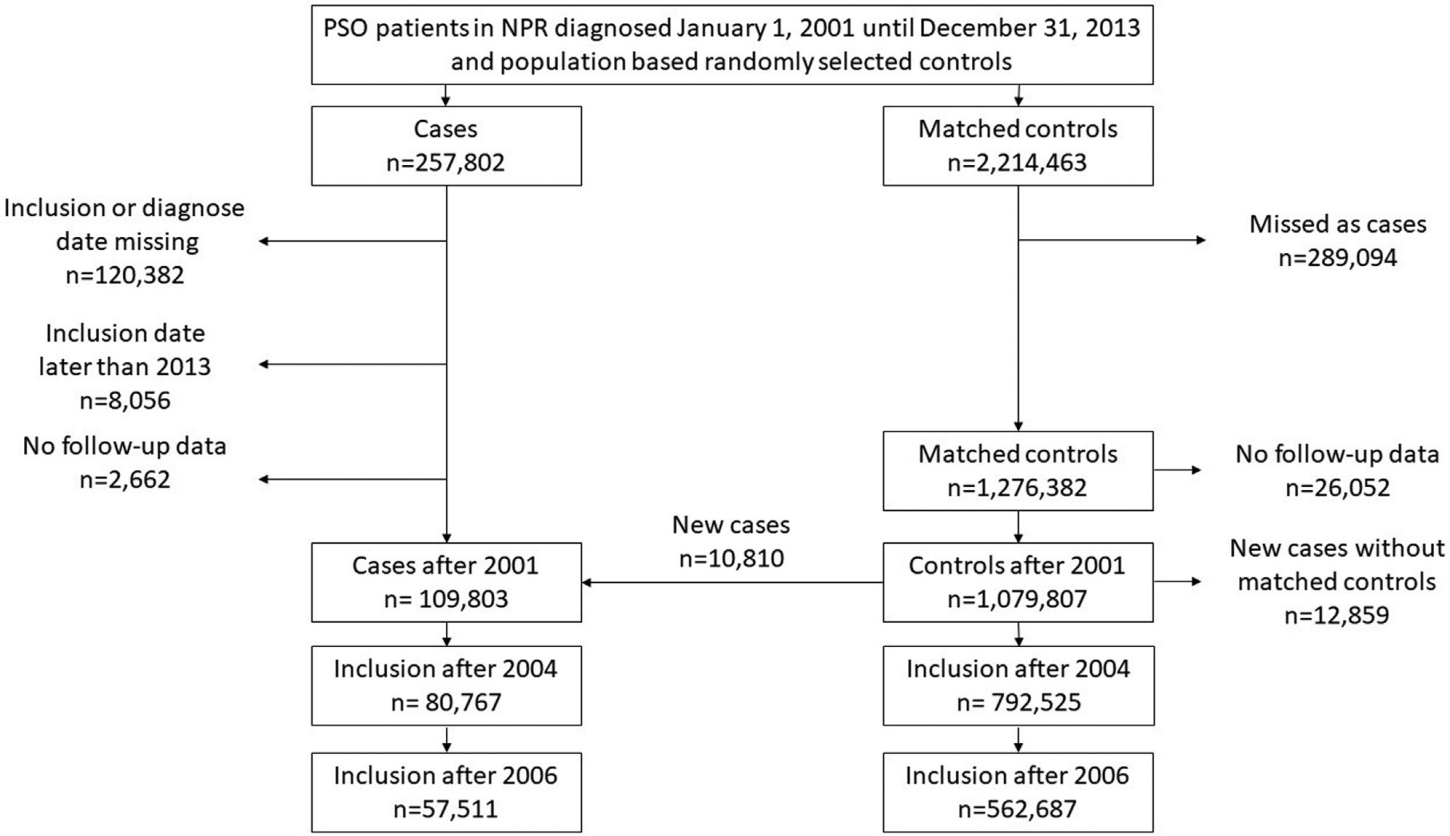
Psoriasis flow chart. *NPR*, National Patient Register; *PSO*, psoriasis.

The study population comprised of all individuals with a first diagnosis of psoriasis, including psoriatic arthritis, in the NPR from 2001 until 2013. A reference cohort was randomly sampled from the TPR (Fig 2). Individuals in the reference cohort are henceforth referred to as controls. All subjects were followed until death, emigration, or end of study (December 31, 2016).

#### Case cohort

Cases were identified from the NPR as patients experiencing at least 1 inpatient or outpatient hospital event for psoriasis (ICD-10: L40) or psoriatic arthritis (ICD-10: M070, M073). The earliest encounter with such diagnosis was the index date. Patients with at least 1 diagnosis of psoriatic arthritis during the first year after diagnosis were classified as having psoriatic arthritis; else they were classified as having psoriasis.

#### Reference cohort

The controls were randomly sampled from the TPR and matched 1:10 to the cases on year of birth, sex, and county of residence. The controls had no diagnosis of psoriasis or psoriatic arthritis prior to the index date. The controls were censored at diagnosis of psoriasis or psoriatic arthritis if that occurred during follow-up (later than December 31, 2013).

### Outcomes studied

Outcomes were education (time to first increase in level of education), individual income, employment status, and marital status, as well as health care and drug utilization. All were recorded/calculated on a yearly basis.

Education was classified into three levels according to the Swedish educational system. Low education was ≤9 years (primary school), medium education was 10-12 years (secondary school), and high education was >12 years (college/university). Income was yearly individual income in Swedish crowns (SEK), 1SEK = 0.1 USD (December 2019). Employment status corresponded to employed or unemployed. Marital status was evaluated as proportions of marriage or registered partnership.

Health care utilization was measured as the number of outpatient visits and hospitalizations. Drug utilization was measured as the yearly number of unique substances per patient. Utilization of some pharmacological groups is also presented (Table II), selected as recommended for psoriasis treatment or as common medicines used in ambulatory care.^22^

**Table II:** Sociodemographic characteristics at baseline and health care consumption, diagnoses, and prescription drug utilization during the year before the index date for patients with psoriasis (cases) and reference cohort (controls)

Characteristics	Measure	Psoriasis (cases) n (%)	Controls n (%)
Age	18-29	12,874 (12)	128,045 (12)
	30-64	72,317 (66)	711,505 (66)
	65+	24,612 (22)	240,257 (22)
	Mean	51.3	51.2
	Standard deviation	16.5	16.5
Sex	Female	58,517 (53)	633,659 (53)
	Male	51,284 (47)	555,951 (47)
Diagnosis	Psoriasis only	106,318 (97)	N/A
	Psoriatic arthritis (including psoriasis)	3,485 (3)	N/A
Income∗	High	15,239 (19)	157,577 (20)
	Median-high	16,392 (20)	156,531 (20)
	Median	17,024 (21)	155,653 (20)
	Median-low	16,673 (21)	156,143 (20)
	Low	14,660 (18)	158,109 (20)
	Missing beginning 2005	779 (1)	8,512 (1)
	Mean	2,016	2,033
	Standard deviation	3,402	3,288
	Median	1,737	1,734
	IQR	1,163	1,232
Education∗	≤ 9 years	19,643 (24)	189,307 (24)
	9-12 years	38,297 (47)	347,739 (44)
	>12 years	21,573 (27)	238,017 (30)
	Missing beginning 2005	1,254 (1.6)	17,462 (2.2)
Employment∗	Yes	48,475 (60)	478,346 (60)
	No	31,935 (40)	310,381 (39)
	Missing beginning 2005	357 (0.4)	3,798 (0.5)
Marital status∗	In relationship	37,515 (46)	365,103 (46)
	Not in relationship	42,895 (53)	417,037 (53)
	Missing beginning 2005	357 (0.4)	10,385 (1.3)
Number of outpatient visits	0	109,706 (99.6)	107,9312 (99.8)
	1 to 2	451 (0.4)	2,270 (0.2)
	3 to 5	12 (0)	43 (0)
	6 or more	5 (0)	8 (0)
Number of hospitalizations	0	121,053 (96)	1,205,459 (97)
	1 to 2	4,639 (3.7)	34,814 (2.8)
	3 to 5	513 (0.4)	3,353 (0.3)
	6 or more	67 (0.1)	538 (0.0)
Number of ATC codes†	0	8,164 (14)	160,277 (28)
	1 to 2	12,520 (22)	148,084 (26)
	3 to 5	14,763 (26)	121,290 (22)
	6 to 10	13,354 (23)	87,861 (16)
	11 or more	8,729 (15)	45,661 (8)
Comorbidities	Myocardial infarction incl. congestive heart failure	7,025 (5.5)	52,926 (4.2)
	Peripheral vascular disease	7,850 (6.2)	58,243 (4.7)
	Cerebrovascular disease	470 (3.7)	41,260 (3.3)
	Chronic obstructive pulmonary disease	8,799 (6.9)	65,701 (5.3)
	Dementia	481 (0.4)	7,215 (0.6)
	Diabetes (incl. complications)	6,737 (5.3)	46,274 (3.7)
	Peptic ulcers	2,300 (1.8)	18,177 (1.5)
	Rheumatic disease	5,626 (4.4)	23,312 (1.9)
	Cancer and metastatic solid tumor	7,044 (5.6)	62,672 (5.0)
Prescription medicines†	A02 Ulcer drugs	8,396 (15)	59,804 (11)
	A10 Diabetes drugs	3,464 (6)	26,760 (5)
	B01A Antithrombotics	7,765 (14)	65,289 (12)
	C03, C07, C08, C09 Antihypertensives	15,772 (27)	131,187 (23)
	C10 Lipid-lowering agents	7,363 (13)	62,856 (11)
	D07 Corticosteroids, topical	20,807 (36)	37,808 (7)
	D05 psoriasis	98 (0)	370 (0)
	H01 Corticosteroids	153 (0)	1,539 (0)
	L04 Immune suppressants	1,848 (3)	5,097 (1)
	M01A NSAIDs	14,458 (25)	93,810 (17)
	N02 Analgesics	13,187 (23)	97,437 (17)
	N05 N06 Psychotropics	13,754 (24)	106,704 (19)
	R03 Asthma/COPD-drugs	5,601 (10)	42,721 (8)

*ATC*, Anatomical Therapeutic Chemical classification system; *COPD*, chronic obstructive pulmonary disease; *IQR*, interquartile range; *NSAIDs*, nonsteroidal anti-inflammatory drugs.

∗ Missing = The proportion of missing data for cases and controls with index dates from 2005 (n = 80,767 and 792,525, respectively). No data on socioeconomic variables were available for cases and controls with index dates before 2005.

† Only assessed for patients (cases and controls) with an index date starting from 2007 (n = 57,511 and 562,687, respectively)

### Statistical methods

Descriptive statistics are presented as numbers and proportions for categorical variables and means, medians, standard deviations, and interquartile ranges for continuous variables. No statistical hypotheses of differences at baseline were formulated or tested. All statistical models were adjusted for the matching variables and a comorbidity index based on the count of comorbidities at baseline. The models for socioeconomic variables also included baseline education. The time to first increase in education was analyzed using Cox regression. All other outcomes were compared over time, by year, because the socioeconomic variables were updated on a yearly basis, from 1 year before index until the end of follow-up. The corresponding generalized linear regression models were further adjusted for the calendar year and follow-up time. An interaction term for follow-up time and cohort was included for differences between cases and controls over time. Normal regression with log link function was used to model the mean income. Proportions of employment and registered partnerships were compared using logistic regression. Mean number of outpatient visits, hospitalizations, and prescriptions were modeled by negative binomial regression. The two-sided alpha-level was 5%.

Observations between the matched groups were considered independent, whereas observations from the same group were considered dependent; therefore, cluster-robust sandwich estimators were used.^29^^,^^30^ Because of regional correlations and unobserved common patient characteristics, county was incorporated in the correlation structure of the generalized estimation equation. Analyses were conducted using SAS 7.15 (SAS Institute Inc., Cary, NC, USA).

If information on a particular variable was available, patients were assumed to have the factor if there was evidence for its presence (ie, absence of information was taken to mean absence of the condition). The exception was when “missing” was a possible value, in which case the missing value was retained. In general, subjects with missing information were dropped from the analysis. If a variable had a larger extent of missing values the category “missing” was added.

## Results

A total of 109,803 patients were included (mean age 51.2 years, 53% women), matched to 1.08 million controls (Fig 2).

There were minimal differences in sociodemographic characteristics between cases and controls at baseline (Table II). Both cases and controls had similar income and education and both groups were predominantly employed and were not married.

Most cases and controls had neither hospitalizations nor outpatient visits during the year before the index year (Table II). Slightly more patients with psoriasis had at least 1 outpatient consultation (0.4% vs 0.2%) or a hospitalization (4.8% vs 3.6%). Rheumatic diseases were more common among patients in whom psoriasis developed (4.4% vs 1.9%). Patients with psoriasis had higher proportions of comorbidities except dementia (0.4% vs 0.6%). There were differences in drug utilization during the year prior to index with the largest difference for topical corticosteroids (36% vs 7%).

**Fig 3:**
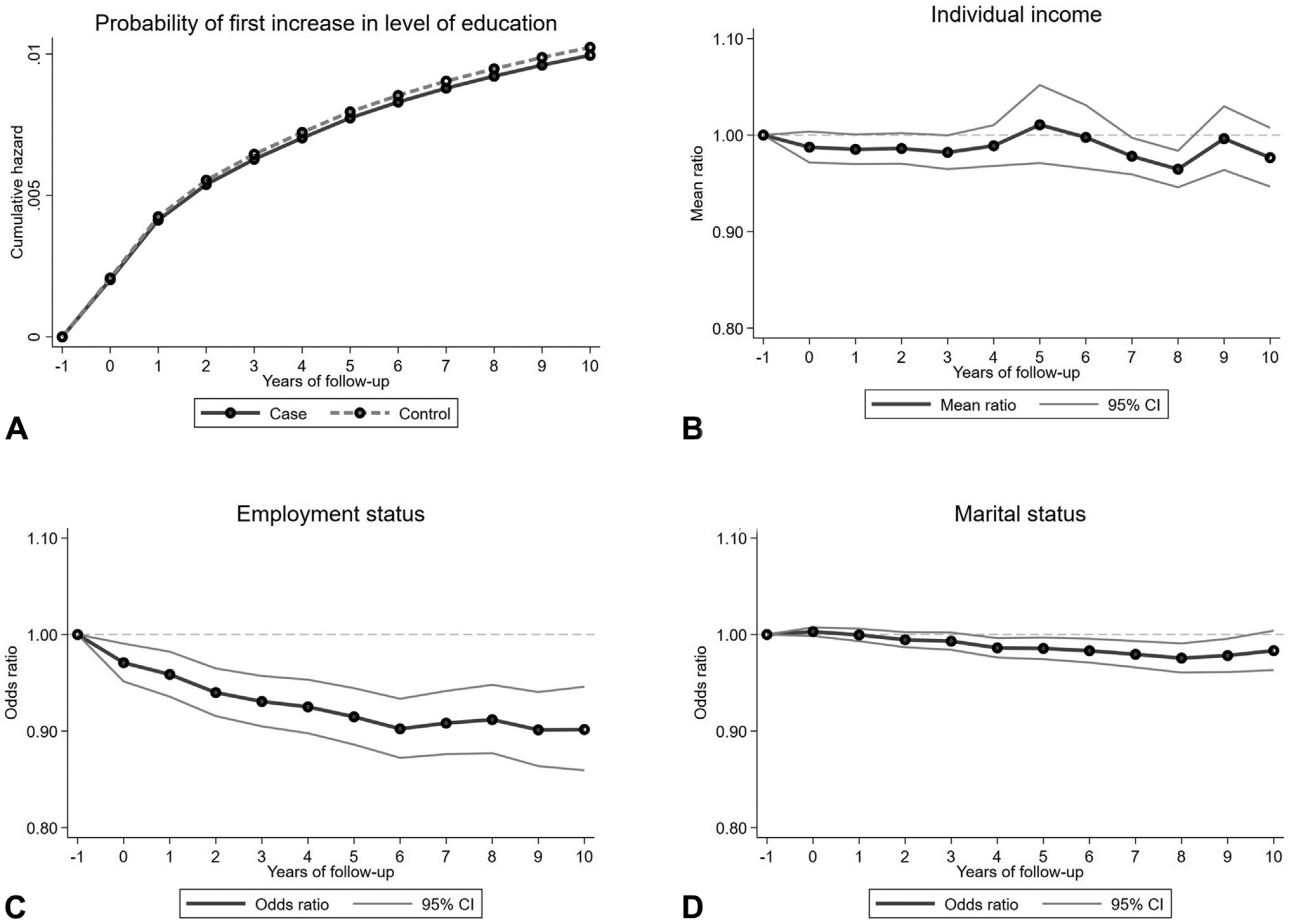
**A**, Psoriasis socioeconomic outcomes over time – level of education. **B**, Psoriasis socioeconomic outcomes over time – individual income. **C**, Psoriasis socioeconomic outcomes over time – employment status. **D**, Psoriasis socioeconomic outcomes over time – marital status. *CI*, Confidence interval.

Changes in socioeconomic status over time for cases compared with controls are illustrated in Figure 3 and listed in Table III.

**Table III:** Odds/mean ratios with 95% confidence intervals and numbers for socioeconomic outcomes for patients with psoriasis (cases) versus reference cohort (controls)

Year after diagnosis	Income					Employment			Marital status		
	Numbers∗		Median		Psoriasis vs controls	Proportion (Yes %)		Psoriasis vs controls	Proportion (Yes %)		Psoriasis vs controls
	Pso	Ctrl	Pso	Ctrl	Mean ratio (95% CI)	Pso	Ctrl	OR (95% CI)	Pso	Ctrl	OR (95% CI)
−1	80767	792525	1737	1734	1 (ref)	60	61	1 (ref)	47	47	1 (ref)
0	80767	792525	1803	1812	0.99 (0.97-1.00)	60	61	0.97 (0.95-0.99)	47	47	1.00 (1.00-1.01)
1	79833	777791	1885	1904	0.99 (0.97-1.00)	59	61	0.96 (0.94-0.98)	47	47	1.00 (0.99-1.01)
2	78753	764313	1962	1996	0.99 (0.97-1.00)	59	60	0.94 (0.92-0.96)	47	48	0.99 (0.99-1.00)
3	77674	751750	2024	2065	0.98 (0.96-1.00)	58	60	0.93 (0.90-0.96)	48	48	0.99 (0.98-1.00)
4	69423	671676	2062	2105	0.99 (0.97-1.01)	57	59	0.93 (0.90-0.95)	48	48	0.99 (0.98-1.00)
5	60643	586774	2093	2142	1.01 (0.97-1.05)	56	59	0.91 (0.89-0.94)	48	49	0.99 (0.97-1.00)
6	52733	510260	2129	2185	1.00 (0.97-1.03)	56	58	0.90 (0.87-0.93)	49	49	0.98 (0.97-1.00)
7	44804	432713	2164	2218	0.98 (0.96-1.00)	55	58	0.91 (0.88-0.94)	49	50	0.98 (0.97-0.99)
8	36812	355410	2179	2245	0.96 (0.95-0.98)	54	56	0.91 (0.88-0.95)	49	50	0.98 (0.96-0.99)
9	28312	273217	2195	2274	1.00 (0.96-1.03)	53	56	0.90 (0.86-0.94)	49	50	0.98 (0.96-1.00)
10	20067	193482	2224	2305	0.98 (0.95-1.01)	53	55	0.90 (0.86-0.95)	50	51	0.98 (0.96-1.00)

*CI*, Confidence interval; *Ctrl*, control; *OR*, odds ratio; *Pso*, psoriasis.

∗ Starting from 2005.

No significant difference (*P* = .38) in time to first increase of education was found between cases and controls (Fig 3, *A*). There was no significant difference (*P* = .99) between cases and controls in income (Fig 3, *B*). There was a tendency toward slightly lower income in patients with psoriasis, with certain variation over time. Significantly lower income was observed at 8 years after diagnosis (*P* = .0003), when patients with psoriasis earned 66,000 SEK less than controls earned.

The largest difference between cases and controls (*P* < .0001) was employment status 2-10 years after index. The proportion of employment decreased among patients with psoriasis compared with that of controls during the first 6 years of follow-up, with 10% less patients with psoriasis being employed (Fig 3, *C*). For marital status, we found a significant difference (*P* < .01) after 4 years of follow-up for the remaining observable period (Fig 3, *D*). The difference in the proportions of registered partnerships was approximately 1.4%.

**Fig 4:**
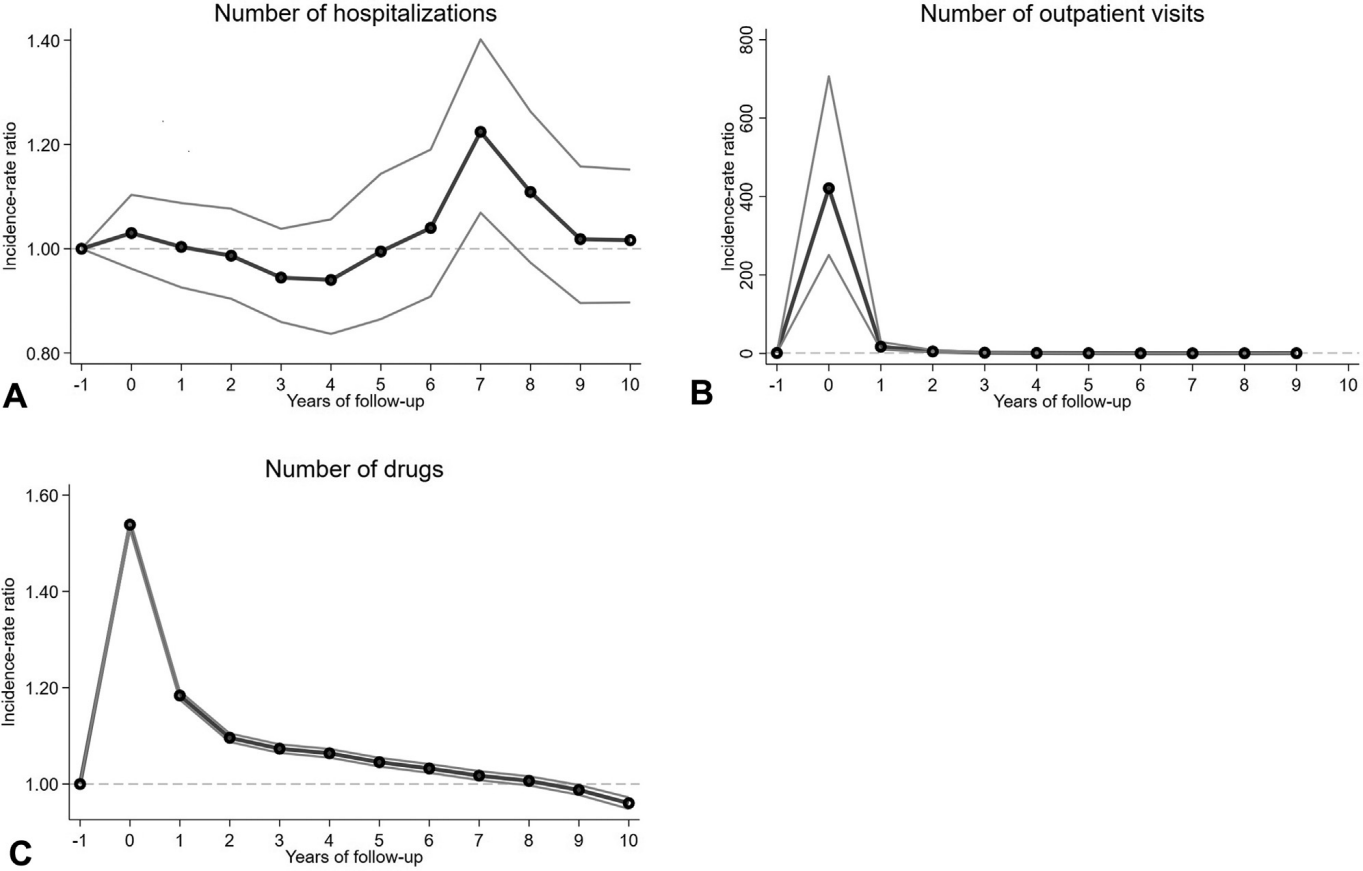
**A**, Psoriasis health care utilization and medicine use – number of hospitalizations. Incidence rate ratios with 95% confidence intervals from negative binomial regression. **B**, Psoriasis health care utilization and medicine use – number of outpatient visits. Incidence rate ratios with 95% confidence intervals from negative binomial regression. **C**, Psoriasis health care utilization and medicine use – number of drugs. Incidence rate ratios with 95% confidence intervals from negative binomial regression.

Health care consumption results are listed in Table IV. The differences in the mean number of outpatient visits (Fig 4, *B*) and unique prescription drugs (Fig 4, *C*) peaked in the year of the psoriasis diagnosis, when the mean difference for patients with psoriasis versus controls were 0.49 and 4.28, respectively. Although the mean number of outpatient visits was only significantly different (*P* < .0001) up to 2 years of follow-up, the mean number of drugs was significantly larger, but with decreasing odds during the first 6 years of follow-up. At 7 years of follow-up, the mean number of hospitalizations was 22% higher for patients with psoriasis than that for controls (*P* = .003).

**Table IV:** Incidence rate ratios and numbers for health care consumption outcomes for patients with psoriasis (cases) versus reference cohort (controls)

Year§	Outpatient visits∗					Hospitalizations†					Drugs‡		
	Number		Mean number		Pso vs Ctrl	Mean number		Pso vs Ctrl	Number		Mean number		Pso vs Ctrl
	Pso	Ctrl	Pso	Ctrl	IRR	Pso	Ctrl	IRR	Pso	Ctrl	Pso	Ctrl	IRR
−1	109803	1079807	0	0	1	0.05	0.04	1	57511	562687	5.47	3.61	1
0	109803	1079807	0.49	0	421.11	0.1	0.08	1.03	57511	562687	8.02	3.74	1.54
1	108730	1062706	0.68	0.03	16.96	0.08	0.06	1.00	56838	552534	6.71	3.83	1.18
2	107306	1045064	0.58	0.05	4.88	0.06	0.05	0.99	56073	543275	5.75	3.51	1.10
3	105788	1027437	0.7	0.16	1.75	0.06	0.04	0.94	55285	534549	5.64	3.48	1.07
4	97045	942353	0.71	0.19	0.96	0.04	0.03	0.94	47345	458096	5.63	3.5	1.06
5	87828	852803	0.72	0.19	0.59	0.02	0.02	0.99	38927	376839	5.4	3.4	1.05
6	79434	771771	0.78	0.22	0.38	0.02	0.02	1.04	31353	303803	5.08	3.23	1.03
7	71041	689735	0.83	0.26	0.26	0.03	0.02	1.22	23729	229722	4.16	2.67	1.02
8	62546	608094	0.95	0.33	0.40	0.03	0.03	1.11	16086	155778	1.67	1.08	1.01
9	53545	521650	1.08	0.4	0.52	0.04	0.03	1.02	7895	76727	N/A	N/A	0.99
10	44815	437642	1.19	0.5	N/A	0.06	0.04	1.02	N/A	N/A	N/A	N/A	N/A

*Ctrl*, Control; *IRR*, incidence rate ratio; *N/A*, not available; *Pso*, psoriasis.

∗ Starting from 2005.

† Starting from 2007.

‡ Starting from 2006.

§ After diagnosis.

## Discussion

In this large cohort, we followed socioeconomic variables and health care consumption for all patients with a first diagnosis of psoriasis in an entire country for more than a decade. We used the comprehensive Swedish registers to address important research gaps. Previous studies on the impact of psoriasis on education and income have either been conducted in small selected populations not representing all socioeconomic groups or with a short follow-up period.

A key finding was the lower proportion of people with psoriasis being employed over time, whereas there was no significant difference in development of education or income. Although statistically significant, the absolute effect size of the difference in employment was small. It is important to point out that the interpretation of observed differences is a common issue with statistics addressing clinical or real-life significance. There was a tendency that fewer patients with psoriasis were married over time. Patients with psoriasis had a high consumption of outpatient care and medicines during the first years after diagnosis, but differences declined over time. The explanation might be the diagnostic work-up close to the diagnosis followed by disease maintenance only. Future studies should consider further expanding this study result.

Differences between the cases and controls were identified already at the time of first diagnosis. Patients with psoriasis had more comorbidities and higher medicine use, reflecting the burden of comorbidity. Such differences between patients with psoriasis and healthy controls are well-known and were not subject to any hypotheses in the current study. A recent overview summarizing evidence from the literature in various populations and settings supported associations between psoriasis and a range of cardiometabolic diseases, gastrointestinal diseases, kidney disease, malignancy, infection, and mood disorders.^31^ In our study, the largest difference in drug treatment was observed for topical corticosteroids, which is not surprising because these are recommended as first-line treatment.^22^ Unfortunately, we could not differentiate by the severity of the disease, but the large proportion being treated before diagnosis may indicate diagnostic delay.

No difference was found for education and income, in contrast with other studies.^8^^,^^32^, ^33^, ^34^ The lack of association for education may be explained by the patient age when psoriasis was diagnosed (mean age 51 years). Sweden belongs to the countries with the highest proportion of people with higher education with median age for attainment of a candidate exam of 28 years.^35^ Lack of association with income may be explained by the Swedish social security system with benefits for all who are ill or disabled as well as for those who are unemployed.^20^

The largest difference observed was for employment with 10% less patients with psoriasis being employed 6 years after first diagnosis, in line with another study estimating productivity loss in patients with psoriasis to around 10%.^3^ The small but significant association between marital status and psoriasis may indicate that there is perceived stigmatization, which may influence the quality of life also for partners, supported by questionnaire studies.^36^^,^^37^ It is, however, important to acknowledge difficulties in analyzing marital status and quality of life relationship because there is limited systematic data to inform this.

Our study provided new information on health care consumption and drug utilization patterns in patients with psoriasis. Although patients with psoriasis undoubtedly have higher use of health care when compared with controls, especially at the time of onset, there was some indication of significantly increased rates of inpatient utilization at 5 years of follow-up.

Furthermore, although there was an uptick in outpatient and prescription drug utilization within 1 year of onset, our findings suggest no difference in drug utilization between cases and controls 7 years after onset. This can have important policy implications as it relates to the duration of monitoring patients with psoriasis.

One study strength is the long observation period, because the Swedish setting allows for longitudinal observation of at least a decade. Few settings outside Nordic countries can observe patients in an entire country for such a long time. Nationwide coverage including all patients in Sweden with specialist care diagnosis of psoriasis, or psoriatic arthritis, is also a major strength, especially when adding controls. Another study strength is the national registers allowing for completeness and accuracy of the linkage through the unique personal identity number. A limitation of the study is its generalizability outside the Nordic region, where there are similar populations and health care systems. Another limitation is the lack of primary care diagnoses and lack of early treatments because of the late start (July 2005) of the PDR. In this study, 96.5% of all patients received their first diagnosis in outpatient care and 3.4% in inpatient care. Data from the region of Stockholm^38^^,^^39^ showed that 20% of all ambulatory care consultations between January 2015 and November 2019 in which psoriasis was diagnosed were in primary care (E. Dahlén, personal communication, December 12, 2019). However, most of these patients had at least one consultation in specialist care. In addition, because these are all consultations and not new diagnoses, the impact of not having primary care data was less problematic. Lastly, the variables used were those available in the Swedish national registers, but variables measured more often and/or at a more detailed level might have enhanced the comparisons.

In conclusion, our study extended the understanding of the socioeconomic impact of psoriasis by suggesting lower societal productivity, indicated by reductions in employment proportion, despite similar incomes to those of the control group. Further, the study indicated that there is room for improvement in the management of patients with possible diagnostic delay. It is also important to monitor health care utilization through at least 5 years as prescription drug utilization normalizes to that of healthy controls.

## Conflicts of interest

Dr Linder and Dr Hägg are employees of the Centre for Pharmacoepidemiology, Karolinska Institutet, which receives grants from several entities (pharmaceutical companies, regulatory authorities, and contract research organizations) for performance of drug safety and drug utilization studies. Dr Wettermark is employed by the health region but has an affiliation with the Centre for Pharmacoepidemiology as associate professor. Dr Wennerström is an employee of Janssen and the Department of Epidemiology Research, Statens Serum Institut, Copenhagen, Denmark. Dr Villacorta is an employee of Janssen. Dr Häbel has nothing to disclose.
